# Decoding the genetics of rare disease: an interview with Monkol Lek

**DOI:** 10.1242/dmm.049694

**Published:** 2022-06-28

**Authors:** Monkol Lek

## Abstract

Monkol Lek, Assistant Professor at Yale University School of Medicine, and Associate Editor at Disease Models & Mechanisms, dedicates his research to finding a genetic diagnosis and improving treatments for rare disease patients. As he originally studied computer engineering at the University of New South Wales in Sydney, Australia, he now utilises computational methods to optimise large-scale genetic studies, provide globally accessible resources for genetic research communities and, importantly, resolve diagnostic odysseys for rare disease patients. Monkol completed his PhD in Prof. Kathryn North's lab at the University of Sydney, studying the genetics of muscle strength and performance, and then continued his investigation of muscle disease in Prof. Daniel MacArthur's lab at Massachusetts General Hospital and the Broad Institute. During his postdoc, he led several large-scale studies aimed at distinguishing pathogenic from benign variants, including the Exome Aggregation Consortium (ExAC) project ( Lek et al., 2016). Monkol established his own lab at Yale University School of Medicine, which continues to improve the diagnosis and treatment of rare muscle disease, and also focuses on underserved populations, whose genetic mutations are not as well characterised as those of European ancestry. In this interview, Monkol discusses how his own diagnosis with limb girdle muscular dystrophy has shaped his career and what he envisions for the future of genetic research in rare disease.


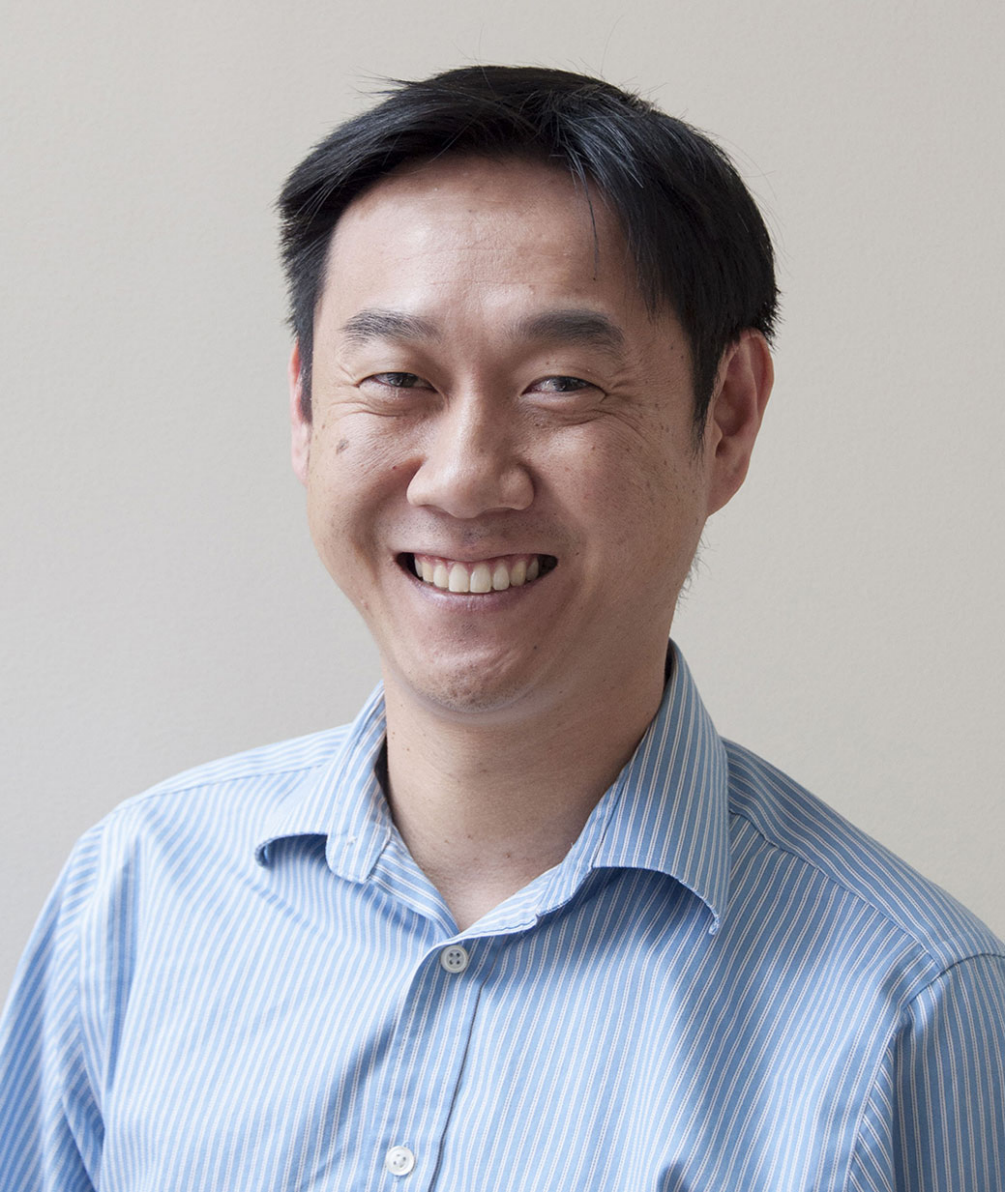


**You have a very unique career path – could you tell us a little bit about that?**

My first degree was in computer engineering. When I first went to university, I studied the hardware and software of computers. I really liked the software aspect of the degree, and so I worked for IBM as a software developer when I finished university. However, during the last few years of university, I noticed that my muscles were getting weaker. My university was on a big hill, with classes at the bottom and top of the hill, and I had to stand up for about 3 h a day while commuting on public transport. It started becoming obvious that I had something wrong with my muscles because I felt totally exhausted at the end of the day. It was frustrating, because I felt that my performance at university was impacted by something that had nothing to do with my ability to think. So, I went from doctor to doctor to try to find out what was wrong with me. As a lot of doctors are not trained in rare diseases, they didn't consider a rare disease diagnosis. Then one doctor did a blood test for creatine kinase (CK), which is leaked into the bloodstream when muscle is damaged. In healthy people, high levels of CK are detected in the bloodstream after they've done intensive exercise, like a marathon. If someone hasn't done something like that, but they have high levels of circulating CK, it could be an indication that there's something wrong with their muscles. As I had high levels of CK in my bloodstream, I then went to a neurologist, which was when I got a clinical diagnosis. At that point, they didn’t know the root cause of the problem, but they knew that I have a muscle disease based on several tests, including a nerve conduction test.

I received this clinical diagnosis during my time in IBM, and that's when I became dissatisfied with my job, because I felt that I was using all my talents to make a very big, international company richer. I was also becoming frustrated when visiting the neurologist every 6 months, as all they would tell me was that my muscles were getting weaker, which I already knew. I began to think that not much was happening in the neuromuscular disease field if that's the best they could offer me. I wanted to know what the root cause of my disease was and if there were any treatment options. I came to the conclusion that no one would care about my disease more than I would, because I'm the one that has lived with it every day of my life.

That's when I decided to leave IBM and pursue a career in researching muscle disease. It didn't go down well with my parents and friends, because I was leaving a well-paid job to go back to university to get paid nothing for an unknown number of years. If I had known my chances of success – completing a meaningful PhD, doing a meaningful postdoc and landing a faculty position – I wouldn't have gone on this journey. I have been very fortunate, but I wasn't always in the right place at the right time.

When I finished my undergraduate degree in bioinformatics and physiology at the University of New South Wales, I started a PhD in Melbourne, but it didn't work out, because not all supervisors are perfect. My wife and I then returned to Sydney, where my wife bumped into one of the professors from our undergraduate degree. She explained that we'd had a bad experience in Melbourne with our PhDs, but our passion was still to do muscle research. The professor's daughter was researching muscle disease in Kathryn North's lab at the University of Sydney, and she invited us to visit the lab. I was offered an opportunity to do my PhD in Kathryn's lab, but I was initially reluctant as it was a diagnostic lab, and I was more interested in developing therapies for people with muscle disease. However, I thought I could still learn a lot about muscle physiology and, in the long term, I'm glad that I received training and mentorship from Kathy's lab. Also, if I hadn't done my PhD there, I wouldn't have met Daniel MacArthur, my future boss. He was a very talented student in Kathy's lab, who taught me a lot about scientific communication among other things, and I taught him some coding skills. He left to work on the 1000 Genomes Project in Cambridge, UK, but I kept in contact with him to get his advice on my project.

When I was finishing my PhD, Daniel asked if I wanted to join the lab he was setting up in Massachusetts General Hospital and the Broad Institute. His lab was going to study common loss-of-function mutations in human populations using large datasets from the 1000 Genomes Project, but he offered me a project investigating neuromuscular diseases. As soon as I submitted my PhD thesis, I started working in his lab. This was perfect timing, because it was 2012, when exome sequencing had recently been published in the context of rare diseases (Ng et al., 2010) and, more importantly, it was becoming affordable, in terms of research. I waited over 10 years for a genetic diagnosis, so my goal was that no one should have to wait that long in the future.

Through collaboration with our former PhD lab, Daniel and I used samples from undiagnosed patients to find answers for Australian families. The first family had two affected girls with undiagnosed nemaline myopathy, who had been on a diagnostic odyssey for about 9 years. It was amazing how quickly we progressed from receiving the samples to identifying the novel gene, *LMOD3*, associated with their disease (Yuen et al., 2014). This was part of my main project during my postdoc – working on gene discovery in neuromuscular diseases and finding answers for patients that have been waiting years and years to get a genetic diagnosis (Ghaoui et al., 2015; O'Grady et al., 2016).

The project that most people know me for is the ExAC project, which was initially my ‘side’ project during my postdoc. The idea was to create a big database of all rare variants that we see in the general population, so we can better interpret the rare variants that we see in rare disease patients. When we were creating it, we thought that it may be useful to other researchers around the world. Therefore, we tried to ensure, through data-use agreements and consent processes, that we could share as many of our findings as possible. I'm happy to say my side project was quite successful. After that, I led other projects, including an analysis group in the Centre for Mendelian Genomics, to expand that framework and idea across all rare diseases, not just neuromuscular diseases (Baxter et al., 2022).

I was having a lot of fun at the Broad Institute, and I was co-author on a lot of high-impact papers. However, the reason I left the Broad Institute was that I wanted to be involved in the full journey for the patients. Sometimes scientists don't understand that getting a genetic diagnosis is not the end of the journey for a patient. After the diagnosis they want to know what treatment options are available. Yale gave me the opportunity to continue doing the gene discovery and analytical work that I was doing at the Broad Institute, plus the capability of doing experiments with mouse models to investigate gene replacement therapies and other therapeutic approaches.

“I waited over 10 years for a genetic diagnosis, so my goal was that no one should have to wait that long in the future.”

**How has being both a researcher and a patient affected your career?**

When I was first diagnosed, there was a neurologist who discouraged me from researching my own disease and this became the basis of my TEDx talk, because I thought it was very condescending. I thought, “Just because I have this disease, it doesn't mean that I have a low IQ”. However, this experience motivated me more. I discussed it with Kathy before starting my PhD, and her encouragement and enthusiasm was refreshing. At the time, in the early 2000s, people hadn't accepted the idea of patients researching their own disease. Things have changed since then, mainly because there are more examples of it now (Branca, 2019), but at the time, it was really hard for me to progress in science. I always thought that people were looking at me with sympathy, and I felt like I had to achieve twice as much to get the same respect as someone else who wasn't as talented or didn't work as hard as me. It was frustrating, but in everyday life people still correlate physical disability with intellectual disability. For example, if my wife is pushing me in the wheelchair in public, no one ever directs a question to me because they assume that the physical disability comes with mental disabilities. There are well-known examples of scientists with physical disabilities, like Stephen Hawking, but it is still challenging in academia when you have a physical disability and people make certain assumptions about you.

On the other hand, just before starting at Yale, my collaborators at the University of Massachusetts took a skin biopsy from me. With this skin biopsy, they created induced pluripotent stem cells, and, using CRISPR, they corrected my disease-associated gene variant in the cultured cells. They then published this in a *Nature* article, in which fig. 1 is the experiment in which they corrected my mutation (Iyer et al., 2019).

**Are there specific skills or knowledge you learned while working in computer engineering that have helped shape and develop your research today?**

When I started my PhD, there was an increase in how much genetics research, and biological research in general, relied upon big data. It can be very challenging to work with big data if you're a biologist without a background in computer science. You can go online to teach yourself to an extent, but it gives you an advantage to learn the theory behind a lot of algorithms and other aspects of software engineering, in a formal setting. It makes the difference between building tools that take a week to analyse a set of data and building tools that take a few minutes to analyse the same data. If you can analyse the data more quickly, you can explore different possibilities and ideas much more quickly. You can't learn everything online, and having a firm foundation of knowledge can enable you to work with big data in an efficient way.

The other thing that you learn from computer science is a certain mindset when approaching problem solving. This is because you have to debug code frequently and, due to this fast pace, you learn quickly. This helped me to troubleshoot problems in biological research quickly.

“Getting a genetic diagnosis is not the end of the journey for a patient. After the diagnosis they want to know what treatment options are available.”

**What do you think are the key challenges for rare disease research and diagnosis moving forward?**

I now have a greater appreciation of the challenges because I see it from two points of view: one as a researcher in a group and one as a PI, who leads the research. The diagnosis rate for rare disease is about 50%, so there are still 50% of patients with a disease that has an unknown genetic cause. The gold standard requirement for associating a new disease gene with a novel phenotype is that it presents in multiple unrelated families (MacArthur et al., 2014). However, when you work with rare diseases, there is the issue of small sample numbers. One challenge for basic scientists is creating good collaborations with physician scientists across the world to enable you to create a large enough dataset.

The other challenge is the cost of research for these diseases with unknown genetic cause. The 50% of cases for which we know the genetic cause are no longer considered an area of research, as clinical genetic services can now diagnose these patients. To diagnose the remaining patients, you have to use more expensive technologies, such as long-read sequencing.

The last thing is the interpretation of rare variants. Although the ExAC project helped with this, there is still a challenge. For example, if a patient has a rare genetic variant, this doesn't necessarily mean it is the cause of their rare disease. This is because even healthy people have rare variants. So, we have a massive interpretation challenge in rare disease genetics, which can be overcome by creating a laboratory model system with that genetic variant to investigate it further. However, if you had 1000 variants to consider, it's not going to scale as an animal model. So, an important question is how can we interpret these variants in a scalable manner? This is one of the main driving forces behind the new Subject Focus, ‘Genetic variance in human disease: decoding diversity to advance modern medicine’, that we are launching in DMM.

**You have led and coordinated several studies involving very large cohorts. From your experience what are the key components of a successful study?**

I think the key to a successful large cohort study with unsolved rare disease patients, is the amount of structured phenotype data you can collect. This requires a good collaborator, who has the time to prepare that data in a meaningful way, which makes it easier to find other families with the same rare disease. The other thing is to have the ability to recontact patients and collect different samples from them, because we're moving to a more multi-omics world. Therefore, we need the ability to go beyond just collecting DNA samples. Also, we're in a world where we're starting to link data to electronic health records, which allows the collection of deeper and richer phenotype data that enable associations to be made between families.

In addition, you can't work in isolation. In order for us to make a meaningful impact, we need to work with groups that have specialties outside of our own. For instance, we collaborate with groups that specialise in the interpretation of non-coding variants. This is important as variants in these regions could hold the answers for some of those unsolved cases.

Another key aspect to a successful study is collaboration with statistical geneticists because some of the more complicated questions are best asked by them. Some of these questions go beyond monogenic diseases. We are seeing convergence between genome-wide association studies, looking for many variants, each with very small contributions to a disease, and studies of Mendelian disease that are looking for one gene that causes disease. The field has to start looking at diseases in the middle of this spectrum, which requires statistical geneticists. This is because you need to make sure that your conclusions are correct. For instance, if you're asking whether a rare disease is caused by a combination of two genes, then you must have a robust statistical model to show that these variants aren't presenting together by chance. You have to prove that those two variants are acting in concert, instead of independently, to cause this disease. My colleagues at Yale published a great paper that demonstrated this concept (Timberlake et al., 2016).

Lastly, it is important to forge meaningful collaborations beyond academia. A lot of my colleagues are being funded by industry collaboration, and a lot of these companies have access to more samples than we do in academia. You can also collaborate with large biobanks, such as the UK Biobank, which has a rich set of phenotype data and also the ability to recontact patients (Glynn and Greenland, 2020). The FinnGen project is a recent public–private collaboration that combines genetic data with electronic health records from Finnish biobank participants to improve disease diagnosis and treatment (Kurki et al., 2022 preprint). So, working with biobanks and industry is another way of increasing sample numbers, which is the biggest challenge in rare disease research.

“We don't want to create disparity in terms of health, especially in the context of genetics, which will continue to become more prominent in modern medicine.”

**You dedicate a lot of your research towards patients in underserved populations, such as East Asian populations, whose genetic mutations are not as well characterised as those of European ancestry. Can you explain the importance of this?**

One of the reasons that it took over 10 years for me to get a genetic diagnosis was because the gene that causes my disease was first reported as not commonly associated with disease in populations of European ancestry. The problem with biomedical research is that when people read that, they think it applies to everyone, even patients who have non-European ancestry. Although the gene that causes my disease aligned with my muscle disease phenotype, it wasn't sequenced because of this assumption. They only decided to sequence this gene once they did linkage analysis of my family, and this was the only gene associated with neuromuscular disease in the linkage region they identified. This is the reason why we need to have good data on all populations. The ExAC and gnomAD studies that I worked on acknowledged that we need good allele frequency data for populations of East Asian, South Asian, Latino and African ancestry, because we don't want to create disparity in terms of health, especially in the context of genetics, which will continue to become more prominent in modern medicine.

If you want to deliver the best healthcare, you have to realise that some variants and diseases are more common in certain populations, such as Tay-Sachs disease, which is common amongst the Jewish community, and sickle cell anaemia, which is more prevalent in populations of African ancestry. By understanding these differences, we can actually find a genetic diagnosis a lot quicker. If it's not a *de novo* variant, and is instead a variant inherited in the population, and if you've made the discovery in East Asians, there is a better chance of identifying more incidences of this variant in the population in which it was first discovered.

I think it's also good for validation of data, because if you had discovered a potential disease-causing variant and you find that this variant has a frequency of 1% or higher in a non-European population, then it's impossible for it to be the cause of a rare disease, regardless of its frequency in a European population (Lek et al., 2016).

## References

[DMM049694C1] Baxter, S. M., Posey, J. E., Lake, N. J., Sobreira, N., Chong, J. X., Buyske, S., Blue, E. E., Chadwick, L. H., Coban-Akdemir, Z. H., Doheny, K. F. et al. (2022). Centers for mendelian genomics: a decade of facilitating gene discovery. *Genet. Med. Off. J. Am. Coll. Med. Genet.* 24, 784-797. 10.1016/j.gim.2021.12.005 PMC9119004 35148959

[DMM049694C2] Branca, M. A. (2019). Their lives in their hands. *Nat. Biotechnol.* 37, 1255-1260. 10.1038/s41587-019-0303-z 31690883

[DMM049694C3] Ghaoui, R., Cooper, S. T., Lek, M., Jones, K., Corbett, A., Reddel, S. W., Needham, M., Liang, C., Waddell, L. B., Nicholson, G. et al. (2015). Use of whole-exome sequencing for diagnosis of limb-girdle muscular dystrophy: outcomes and lessons learned. *JAMA Neurol.* 72, 1424-1432. 10.1001/jamaneurol.2015.2274 26436962

[DMM049694C4] Glynn, P. and Greenland, P. (2020). Contributions of the UK biobank high impact papers in the era of precision medicine. *Eur. J. Epidemiol* 35, 5-10. 10.1007/s10654-020-00606-7 31993883

[DMM049694C5] Iyer, S., Suresh, S., Guo, D., Daman, K., Chen, J. C. J., Liu, P., Zieger, M., Luk, K., Roscoe, B. P., Mueller, C. et al. (2019). Precise therapeutic gene correction by a simple nuclease-induced double-stranded break. *Nature* 568, 561-565. 10.1038/s41586-019-1076-8 30944467 PMC6483862

[DMM049694C6] Kurki, M. I., Karjalainen, J., Palta, P., Sipilä, T. P., Kristiansson, K., Donner, K., Reeve, M. P., Laivuori, H., Aavikko, M., Kaunisto, M. A. et al. (2022). FinnGen: Unique genetic insights from combining isolated population and national health register data. *medRxiv*. 10.1101/2022.03.03.22271360

[DMM049694C7] Lek, M., Karczewski, K. J., Minikel, E. V., Samocha, K. E., Banks, E., Fennell, T., O'Donnell-Luria, A. H., Ware, J. S., Hill, A. J., Cummings, B. B. et al. (2016). Analysis of protein-coding genetic variation in 60,706 humans. *Nature* 536, 285-291. 10.1038/nature19057 27535533 PMC5018207

[DMM049694C8] MacArthur, D. G., Manolio, T. A., Dimmock, D. P., Rehm, H. L., Shendure, J., Abecasis, G. R., Adams, D. R., Altman, R. B., Antonarakis, S. E., Ashley, E. A. et al. (2014). Guidelines for investigating causality of sequence variants in human disease. *Nature* 508, 469-476. 10.1038/nature13127 24759409 PMC4180223

[DMM049694C9] Ng, S. B., Buckingham, K. J., Lee, C., Bigham, A. W., Tabor, H. K., Dent, K. M., Huff, C. D., Shannon, P. T., Jabs, E. W., Nickerson, D. A. et al. (2010). Exome sequencing identifies the cause of a mendelian disorder. *Nat. Genet.* 42, 30-35. 10.1038/ng.499 19915526 PMC2847889

[DMM049694C10] O'Grady, G. L., Lek, M., Lamande, S. R., Waddell, L., Oates, E. C., Punetha, J., Ghaoui, R., Sandaradura, S. A., Best, H., Kaur, S. et al. (2016). Diagnosis and etiology of congenital muscular dystrophy: We are halfway there. *Ann. Neurol.* 80, 101-111. 10.1002/ana.24687 27159402

[DMM049694C11] Timberlake, A. T., Choi, J., Zaidi, S., Lu, Q., Nelson-Williams, C., Brooks, E. D., Bilguvar, K., Tikhonova, I., Mane, S., Yang, J. F. et al. (2016). Two locus inheritance of non-syndromic midline craniosynostosis via rare *SMAD6* and common *BMP2* alleles. *eLife* 5, e20125. 10.7554/eLife.20125 27606499 PMC5045293

[DMM049694C12] Yuen, M., Sandaradura, S. A., Dowling, J. J., Kostyukova, A. S., Moroz, N., Quinlan, K. G., Lehtokari, V.-L., Ravenscroft, G., Todd, E. J., Ceyhan-Birsoy, O. et al. (2014). Leiomodin-3 dysfunction results in thin filament disorganization and nemaline myopathy. *J. Clin. Invest.* 124, 4693-4708. 10.1172/JCI75199 25250574 PMC4347224

